# Emotion Induced Blindness Is More Sensitive to Changes in Arousal As Compared to Valence of the Emotional Distractor

**DOI:** 10.3389/fpsyg.2017.01381

**Published:** 2017-08-15

**Authors:** Divita Singh, Meera M. Sunny

**Affiliations:** Centre for Cognitive Science, Indian Institute of Technology Gandhinagar Gandhinagar, India

**Keywords:** emotion induced blindness, arousal biased competition, attentional blink

## Abstract

Emotion Induced Blindness (EIB) refers to the impairment in the identification of a neutral target image that follows a threatening or fearful distractor image. It has been suggested that valence plays a significant role in driving the perceptual impairment in EIB. Recent findings from the literature suggest that arousal has a very important role in biasing early cognitive functions. Hence, in the present study, we systematically investigate the role of valence (Experiment 1) and arousal (Experiment 2) in determining the impairment in EIB. The results suggest that when valence is controlled for, the stimuli with higher arousal level lead to greater impairment in target detection. Moreover, under high arousal condition, both positive and negative stimuli lead to significantly greater impairment in target detection. Present study suggests that impairment in EIB is sensitive to the arousal component of the emotional image as compared to valence. The arousal biased competition account that explains the effect of arousal on cognitive processing can sufficiently explains the current results.

## Introduction

Many recent studies have shown that attentional resources are allocated rather automatically (bottom-up attentional allocation) and efficiently to emotional stimuli as compared to non-emotional stimuli (Carretié et al., 2004; Phelps et al., 2006; Yiend, 2010; Pool et al., 2015). This is not very surprising considering that emotional stimuli usually indicate relevance and their efficient processing would be generally advantageous (Öhman, 1986; Taylor, 1991; Panksepp, 1998; Alpers and Gerdes, 2007; Bublatzky et al., 2014).

Neverthless, the specific mechanism by which emotional stimuli are automatically processed is long standing and controversial. Researchers have argued that valence is critical in understanding how emotional stimuli are processed while others have emphasized the role of arousal. That is, on one hand, researchers have shown that negatively valenced stimuli enjoy attentional priority over neutral and positive stimuli (Pratto and John, 1991; LeDoux, 1996; Öhman et al., 2001; Vuilleumier et al., 2001; Anderson et al., 2003; Watson et al., 2011, 2012; Yang et al., 2016), while on the other hand, researchers have argued that arousal plays an important role in the processing of emotional stimuli (Kensinger, 2004; Schimmack and Derryberry, 2005; Kensinger and Schacter, 2006; Lang and Bradley, 2010; Mather and Sutherland, 2011).

In the present study, we aim to see the role of emotionality of a task irrelevant emotional image (distractor) in determining the impairment in the detection of a subsequent neutral target image. Most et al. (2005) refered to this effect as Emotion Induced Blindness (EIB). A typical Emotion-induced blindness task involves rapid serial visual presentation (RSVP) of images, at the rate of 100 ms each and task of the subject is to report the single target image (neutral) which is preceded by emotional or neutral image (distractor). Target can appear at various time periods, typically between 100 and 800 ms (lag1–lag8) after the presentation of the emotional distractor. It is observed that when target appears near to the emotional distractor, i.e., between 100 and 500 ms (lag1–lag5) after the appearance of the emotional image, accuracy was very low. However, accuracy improved when the duration between the emotional distractor and target was larger than 500 ms.

Most et al. (2005) argued that the impairment associated with target processing is perceptual in nature and that the effect is emotion specific. Emotion specificity in this context means that the perceptual impairment is attributed to the emotional nature of the distractor and to the mechanisms involved in the processing of the emotional stimuli. Many EIB studies emphasize the role of negative valencein driving EIB. For example, Most et al. (2005) showed that when positively valenced stimuli are used as emotional distractor, EIB is not observed. However, unlike previous researchers, we are interested to see if the EIB effect is more senstive to the valence or arousal component of the emotional picture.

So far, the focus has been to understand what discrete emotional categories lead to the perceptual impairment of a succeeding neutral target. For example, Ciesielski et al. (2010) showed that as compared to neutral images, fear, disgust, and erotic images showed larger impairment for a subsequent neutral target image. Additionally this impairment was greater for erotic images as compared to fear and disgust category of distractor images. Similarly, Most et al. (2007) showed that erotic images leads to impairment in the detection of subsequent targets, compared to non-erotic images. It is interesting to note that all the three categories (Fear, Disgust, and Erotic) of pictures would also be high in their arousal component (Lang et al., 2008). However, Ciesielski et al. (2010) do not differentiate between the role of valence and arousal in the magnitude of the impairment. Nevertheless, this seems implicit in their findings where they do not find any differences between the various emotional categories.

However, they have not considered or controlled for the level of arousal of the emotional stimuli. Also, in some cases, it is not clear whether arousal or valence is critical in determining the observed impairment in target detection. For example, Ciesielski et al. (2010) found EIB when the emotional distractor was erotic in nature. However, they argued that these are negatively valenced as there is some shock-value associated with erotic images. However, there is no strong evidence to support the claim that erotic images are negatively valenced.

Further insight into the role of arousal and valence in determining the impairment observed in EIB can be gained from the finding of Arnell et al. (2007). They used an Attentional Blink (AB) paradigm to show that arousal, rather than valence of the emotional word is a better predictor of the impairment in the identification of a neutral target word that follows. They also argued that at high arousal levels, both postively and negatively valenced stimuli leads to AB. Arguably, AB and EIB are mechanistically different^1^. AB involves RSVP of alphanumeric lettesrs/images and have generally have two targets (Target1 and Target2) and the typical finding is that Target2 accuracy is severely impiared if it appears between 200 and 500 ms from the target1. This impairment in target2 is refrerred as AB as the impairment in Target2 accuracy is thought to an attentional limitation (Raymond et al., 1992). Neverthless, there is considerable similarity in the behavioral manifestations of AB and EIB and hence, it is possible that the arousal and valence has a similar effect on EIB as it has on AB. Hence, the findings from the present study will also offer a way to check if the emotional arousal and valence have similar effects on EIB as in AB or are they different.

In fact, Arnell et al.’s (2007) task setting is very similar to that of EIB. They only had a single target and the emotional stimuli was functionally a distractor. They showed that an emotional word presented in a RSVP stream can result in the impairment in the detection of a neutral target word even when the emotional word is irrelevant to the task. Even though the specific mechanisms responsible for AB and EIB may be different, it is possible that valence and arousal play a similar role in mediating AB and EIB. If this is the case, one might argue that similar mechanisms mediate the processing of emotional information in AB and EIB.

Hence, the present study has two aims – to investigate the differential effects of arousal and valence in EIB as well as to compare the results with findings from AB. We hypothesized that the arousal level of the emotional image in EIB will have a greater impact on the perception of the neutral target image too. If this is the case, then any stimuli (positive/negative) with a high arousal^2^ rating will impair the perception of a neutral target image. In order to test this hypothesis, in the first experiment, we kept valence level of the emotional image constant while varying its arousal level. The results showed that there was a larger impairment with high arousal as compared to low arousal stimuli even when the mean valence of both sets of images was kept constant. In order to see if changes in valence can explain the results, we varied valence while keeping arousal constant (Experiment 2). The results show that at the same valence, both positive and negative stimuli elicit the same EIB. Overall, the results suggest the EIB effect is more sensitive to changes in arousal as compared to changes in valence. This fits with the findings of Arnell et al. (2007), who showed a similar effect of arousal and valence in determining the impairment in AB. The Arousal Biased Competition model (ABC) can very well explain both of these effects (Mather and Sutherland, 2011). ABC model suggest that arousal can bias the cognitive processing in favor of the stimuli by having the competitive advantage over the low arousing stimulus in the early visual (see a more elaborate discussion of ABC model in the general discussion).

## Experiment 1

We presented RSVP of images, which includes emotional distractor images (low and high arousal International Affective Picture System (IAPS) images), target image (neutral; 90°tilted architecture and landscape images) and fillers (upright architecture and landscape images). To test the effect of emotional distractor on target perception over time, the relative position of the target compared to the distractor was also manipulated; target couldappear100 ms (lag1), 200 ms (lag2), or 800 ms (lag8) after the appearance of the emotional distractor. If affective valence is a major factor in determining perceptual impairment then varying the arousal level while keeping valence constant should have little effect on the perceptual impairment to a neutral target. In contrast, if arousal has a larger effect, then we expect a significantly larger impairment in the identification of the target images in the high arousing distractor condition as compared to low arousing distractor condition.

### Method

#### Participants

Fifteen university students (mean age = 23, 2 female) participated in the study. All of them reported normal or corrected to normal vision and gave informed consent prior to the commencement of experimental session.

#### Apparatus and Stimulus

Participants were seated in a sound attenuated, dimly lit room in front of 19” Dell LCD monitor at a resolution of 1280 × 1024 pixels. The stimulus was presented using Matlab with the Psychophysics Toolbox extensions (Brainard, 1997; Pelli, 1997) on an IBM-PC compatible computer. Participants’ responses were recorded using “Alt,” “Ctrl” keys on a standard keyboard. The stimuli were colored photographs with a resolution of 400 × 300 pixels subtending a visual angle of 11° × 8° at approximately 60 cm distance and were presented on a black background.

#### Procedure and Design

**FIGURE 1:**
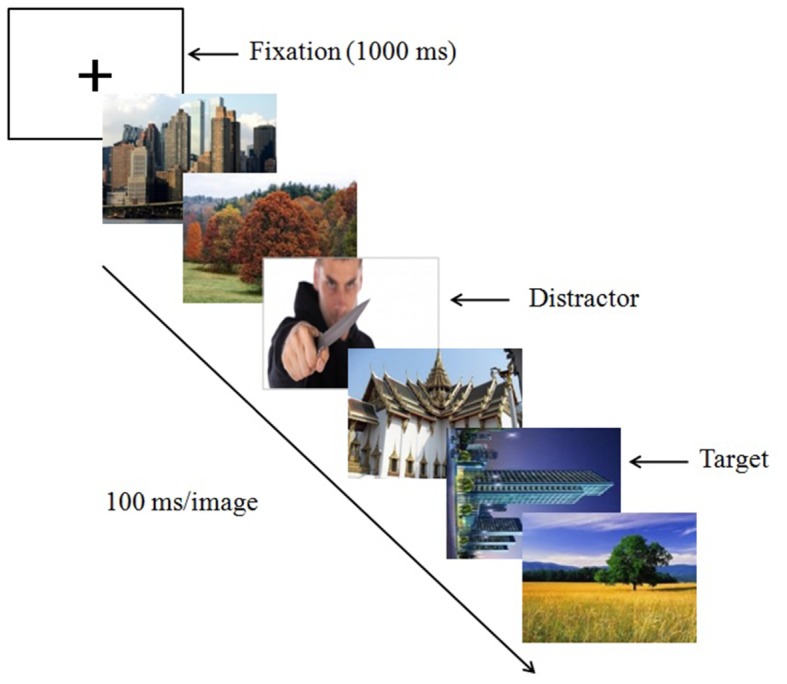
Example figure showing the sequence of RSVP task for Experiments 1 and 2 using an emotional distractor with high arousal and negative valence. The tilted architectural image in this example appears at Lag 2 and is the target.

Each trial starts with the presentation of a fixation cross (1000 ms) on the center of the screen followed by a RSVP of 22 images. Each image was presented for 100 ms without any inter stimulus interval. In each trial, the RSVP stream consists of one emotional distractor, one target (90° tilted to left or right landscape/architecture image) and 20 (or 21 when target is absent) filler images of landscape or architecture (**Figure 1**). The emotional distractors were taken from the IAPS (Lang et al., 2008) whereas the landscape and architecture images were taken from publically available sources. The fillers were selected randomly from a pool of 120 images whereas the target pictures were picked from a different pool of 160 images. The filler images were upright (not tilted) landscape/architectural images that comprise pictures of sceneries, buildings, and houses.

The emotional distractors were selected from a pool of 180 images, half of which were low arousal (mean = 3.5) and half-high arousal (mean = 6.2), but with the same mean valence (mean = 5.5) and standard deviation (1.9). The scrambled images were of scrambled version of high and low arousal emotional images. None of the emotional images were repeated. The emotional distractor appeared at either the 2nd, 4th, 6th, or 8th position of the RSVP stream and the target could appear either at the 1st (Lag 1), 2nd (Lag2), and 8th position (Lag 8) from the emotional distractor. Participants are asked to look for the target, which could be a left or right oriented image. The target was absent in 10% of the trials. The target absent trials acted as catch trials to check if the participants are responding correctly. Once the RSVP stream ended, participants decided whether or not the target was present in the stream by using Y/N keys. If they report that the target was absent (N), then the next trial started after a gap of 1000 ms. If they report having seen the image (Y), they are then asked to report the orientation by using the “Alt” and “Ctrl” for left and right oriented images, respectively. In case they make an error in the orientation judgment task, they got visual feedback on the screen saying “Incorrect response.”

Across four distractor positions and three lags, the target could appear in the 4th, 6th, 8th, 10th, 12th, or 14th position in the RSVP stream. Participants completed 12 practice trials in which no emotional distractor was present and 179 experimental trials, with the trials equally divided between three lags and three distractors conditions. All possible factor combinations were presented in a random order. That is, the experiment systematically varied three factors, Lag (1, 2, and 8) and Arousal (Low, High, and scrambled images) of the emotional distractor while keeping valence constant. Accuracy was measured as an indicator of EIB and included the conditions in which participants reported that they have seen the target image and was accurate about the target orientation.

### Results

Accuracy for target absent trials (catch trials) was calculated to check false alarms. Participants made 3% average errors in the catch trials, erroneously saying that they saw the target that was not present.

**FIGURE 2:**
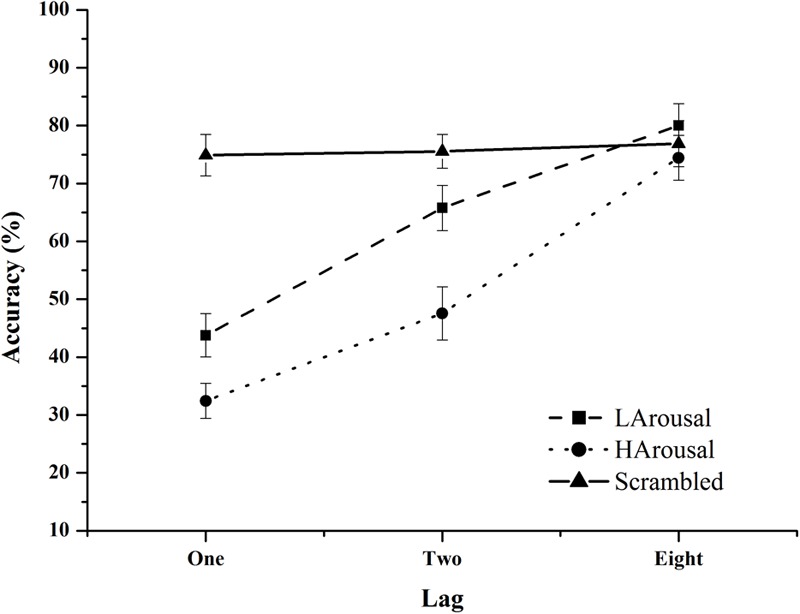
Mean percentage accuracy in Experiment 1 as function of lag with separate lines for each distractor conditions.

Mean percentage accuracies were calculated for each lag and arousal type separately for each participant (see **Figure 2**). These were submitted to a 3 (low arousal and high arousal and neutral) × 3 (lags: one, two, eight) repeated measure ANOVA. The results showed a significant main effect of Arousal, *F*(2,28) = 49.42, *p* < 0.001, $\eta_{p}^{2}$ = 0.77, suggesting that accuracy was lower when the target followed a high arousal distractor (mean accuracy = 51.4%) as compared to low arousal distractor (mean accuracy = 63.18%) and neutral distractor (mean accuracy = 75.7%). *Post hoc* showed that all three distractor conditions are significantly different from each other (High arousal mean accuracy – Lag-1 = 32.5, Lag-2 = 47.6, Lag-8 = 76.9; Low arousal mean accuracy – Lag-1 = 43.8, Lag-2 = 65.8, Lag-8 = 80; Scramble image mean accuracy – Lag-1 = 74.9, Lag-2 = 75.6, Lag-8 = 76.9). The main effect of Lag was also significant *F*(2,28) = 146.6, *p* < 0.001, $\eta_{p}^{2}$ = 0.91 with lowest accuracy at lag-1 (50.3%) and Lag-2 (62.9%) and highest at Lag-8 (77.1%). The interaction between Arousal and Lag was also significant *F*(4,56) = 21.18, *p* < 0.001, $\eta_{p}^{2}$ = 00.6. This was because the accuracy of target detection in the neutral condition did not change over lags whereas for both high and low arousal conditions, the accuracies improved significantly as lag increased (all *p* < 0.01).

### Discussion

In Experiment 1 we manipulated the arousal of the emotional pictures while keeping the valence constant in an EIB task. The result showed that despite of the fixed valance, highly arousing emotional distractor causes greater impairment in target detection as compared to low arousing emotional distractors. That is, accuracy in detection of the neutral target was significantly more impaired when the target follows a high arousing emotional distractor image as compared to low arousing emotional distractor image. Additionally, accuracy in neutral condition was better than both high and low arousal condition. This is more or less in line with standard EIB finding where targets that follow emotional distractors have lower identification accuracy as compared to targets that follow neutral images (Most et al., 2005, 2007; Smith et al., 2006; Most and Jungé, 2008; Most and Wang, 2011; Kennedy and Most, 2012, 2015a,b). It is also important to note that our high arousal images were a mix of erotic, fear, disgust, and awe.

The present findings are also supported by AB studies using taboo words. For example, Mathewson et al. (2008) showed that as compared to a positive, negative, or neutral T1, a Taboo T1 word significantly impaired the detection of a neutral T2 word. They showed the same effect of the taboo word even when it was a pseudo-target (i.e., even when participants were asked to ignore the taboo T1 word and to report only neutral T2 word). That is, even when the participants were not required to attend to the taboo words, it still impaired the perception of a following neutral target word. They also showed that this effect is dependent more on the arousal of the taboo word (Arnell et al., 2007), and less on the valence. Valence affects perceptual impairment only for stimuli whose valence ratings fall on the extremities of the scale (both highly positive and negative valence).

Importantly, we have added to the literature on EIB and showed that perceptual impairment is observed in the detection of neutral stimuli that is followed by high arousal stimuli even when the valence of the pictures is controlled for. Overall, the finding point to a significant role of arousal in the perceptual impairment associated with EIB as compared to valence and is supported by evidence from AB studies showing similar effects.

Moreover, the current findings are also in line with the general literature that suggest that arousal and valence affect cognitive mechanisms differently. For example, it has been suggested that higher arousal has more impact on automatic processes whereas valence influences controlled processes in memory (Kensinger and Corkin, 2004). Consequently, it could also mean that the impairment in target detection in EIB is a result of impairment in automatic early level processing driven by arousal. Nevertheless, the current study cannot differentiate between the role of controlled and automatic processes in EIB, but suggests that this should be systematically tested in the future. In fact, Most et al. (2005) argues that emotions impair early level visual processing, leading to EIB. However, they did not differentiate the effect of arousal and valence in determining this early impairment.

## Experiment 2

Experiment 2 was conducted to further investigate the differential effect of arousal on EIB for positively valenced and negatively valenced stimuli. In order to test this, we used pictures from the IAPS database that has, on average, same arousal rating (6.4) but different valence. The positive valence stimuli (mean valence rating = 7.6) consists of images that fit into discrete categories of erotic, happy, and awe whereas the negatively valenced stimuli (mean valence rating = 1.8) consists of fear, disgust, and threat. We did not control for the discrete emotions that constitute our positive and negative valence conditions as previous studies have shown that these categories have similar effects on attention (Arnell et al., 2007; Mathewson et al., 2008; Ciesielski et al., 2010). In the present study we expect both positively and negatively valenced emotional distractors to lead to EIB as they are both high in arousal.

Moreover, studies have shown that the effect of arousal on cognitive processes is independent of the valence of the stimuli (Kensinger, 2009). They also suggest the possibility of differential effects of positive and negative valence on perceptual mechanisms. That is, they suggest that while positive valence impairs perceptual processing, negative valence enhances perceptual processing. This will imply a larger impairment in positive valence condition as compared to negative. However, if the valence does not matter, then all of the result will be driven by arousal Overall Experiment 2 is similar to Experiment 1 except that the High Arousal condition was manipulated across two different valence categories (positive and negative).

### Method

#### Participants

Fifteen students (2 female) with the mean age of 23 participated in this experiment. All reported to have normal vision and has given informed consent beforehand.

#### Apparatus and Stimulus

The target and filler images were same as in the previous experiment. Most of the emotional distractors were, however, new and were from the IAPS database. There were three conditions of emotional distractors: Positive (mean valence = 7.4, mean arousal = 6.4), Negative (mean valence = 1.8, mean arousal = 6.4), and Neutral (scrambled version of positive and negative images).

#### Procedure and Design

The procedure and design were similar to that used in Experiment 1 with the following differences. In Experiment 2, the emotional distractors were selected from a different pool of 180 images, half of which were positive valence images and half were negative valence images.

### Results

**FIGURE 3:**
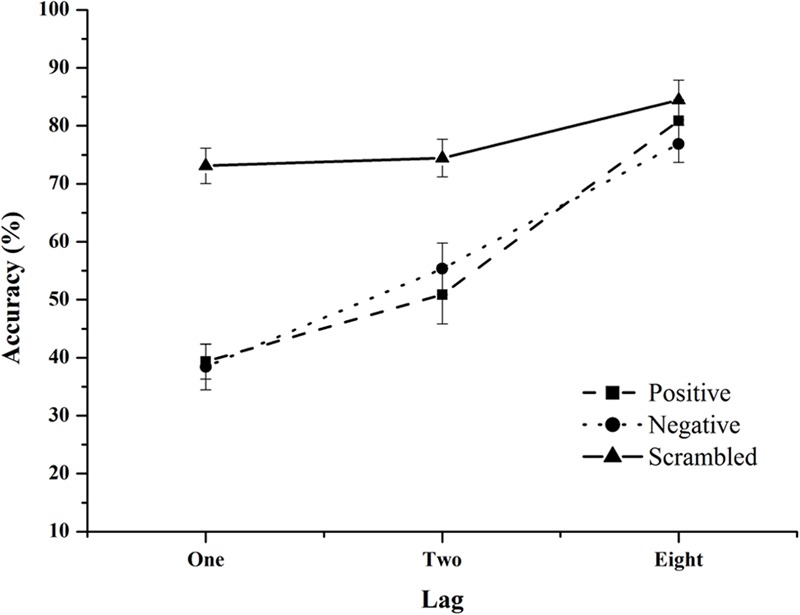
Mean percentage accuracy in Experiment 2 as function of lag with separate lines for each distractor condition.

Accuracy for target absent trials was calculated to check false alarms. Result showed that participants made only 4% errors on average in the target absent category. Mean percentage accuracies were calculated for each Lag and Valence level (see **Figure 3**).

A 3 (Emotion: scrambled, negative, and positive) × 3 (Lags: 1, 2, and 8) repeated measure ANOVA revealed significant main effect of Emotion, *F*(2,28) = 45.12, *p* < 0.001, $\eta_{p}^{2}$ = 0.76. Overall accuracies were significantly different for both negative and positive emotion conditions compared to the scrambled image condition (mean difference of 20.4% and 20.2%, respectively, *p* < 0.001). The overall accuracy was greatest for scrambled distractor condition (77.4%) and lowest for positive (57%) and negative (56.8%) distractor condition. Accuracies in the positive and negative distractor condition were not significantly different from each other, *p* = 0.941. There was also a significant main effect of Lag, *F*(2,28) = 108.7, *p* < 0.001, $\eta_{p}^{2}$ = 0.88. Overall accuracies were significantly different between all three lags (all *p* < 0.001). The greatest accuracy was at lag 8 (80.7%) followed by lag 2 (60.2%) and lag 1 (50.1%). The interaction between distractor type and lag was also significant, *F*(4,56) = 17.4, *p* < 0.001, $\eta_{p}^{2}$ = 0.55. *Post hoc* tests showed that the accuracy of target detection in the scrambled condition did not change over lags whereas for both high and low arousal conditions, the accuracies improved significantly as lag increased (all *p* < 0.01).

### Discussion

Experiment 2 was conducted to examine the relative role of arousal and valence in the perceptual impairment of a neutral target. The mean valence in the high arousal condition was varied to see whether it would have an effect on the detection accuracy in the EIB task. The accuracies in positive and negative valence condition were compared with the neutral condition (scrambled images). The results suggest that target detection was significantly impaired for targets that followed both positive as well as negative distractors as compared to the targets that followed a non-emotional scrambled distractor when their arousal level was controlled for. Moreover, the level of impairment was similar for both positive and negative distractor.

Studies have shown that erotic images often lead to a larger impairment as compared to negative images. For example, Ciesielski et al. (2010) result show that, erotic images showed largest impairment when compared to pictures of disgust and fear. They explained this finding by suggesting that the erotic images have an associated “shock-value” and that this could lead to a larger impairment. The present finding suggests that the difference in the magnitude of impairment might be due to possible differences in arousal inherent in their stimuli. When the arousal is controlled for both positive as well as negative valence leads to similar level of impairment in target detection.

Moreover, the positive images used in the present study contained pictures that were not only erotic but also belonging to other discrete emotional categories such as awe and happy. Hence, the impairment observed in the positive category cannot be well explained by possible negative valence associated with erotic stimuli. Moreover, we used IAPS pictures in which the stimuli are rated as highly positive, making it highly unlikely that the present set of participants experienced shock and thus a negative valence while viewing these pictures. Overall, the results can be explained by changes in arousal and hence a more complicated discrete model might not be necessary to explain the impairment observed in EIB.

Present finding is also consistent with previous studies in which emotionally arousing images have been found to get preferential processing and is a better predictor of attentional bias of the emotional stimuli. For example, Arnell et al. (2007) shows that extremity of the valence, predicts target accuracy but only when it was paired with the high arousal. In the present experiment we only use valence extremes and these are high in their arousal rating. The findings are also in line with that of Schimmack and Derryberry (2005), who showed that arousal significantly predicts variances in response latencies whereas evolutionary threat and categorical negativity theory failed to predict the variances in the data. Similar kind of attentional interference by emotionally arousing pictures can also be seen in Verbruggen and De Houwer’s (2007) study. Using a stop signal paradigm, their results show delayed response latencies in no-go trials and delayed stopping latencies in go-trials for emotionally arousing pictures (both positive and negative) as compared to neutral pictures in a stop signal paradigm. Similarly, McConnell and Shore (2011) shows that participants experiencing a positive valence state had less executive control over their response but only when it was paired with high arousal.

The results from the present experiment is not in line with the predominant narrative within the EIB literature suggesting that EIB is an effect of better processing of evolutionary threat and categorical negativity. For example, Ciesielski et al. (2010) observed less impairment for pictures from the fear and disgust category as compared to the erotic category of emotional images. This finding does not agree with the threat superiority theory. Nevertheless, they suggested that greater impairment for erotic images could be due to the shock-value associated with the erotic images and less impairment for fear and disgust could be due to the hypervision (enhanced performance) for the subsequent target item. However, hypervision, in this case should translate to improved performance at Lag 1 compared to other lags. However, in the present study as well as in EIB generally, the accuracy is worst at the first lag. It is also theoretically harder to explain hypervision extending to items that are not immediately preceding the negative item. Most importantly, they do not consider the role of arousal for this distinct effect when an arousal-based explanation is well supported by the literature. The results of the present study clearly show that positive emotions also lead to strong impairment in target detection as long as they are matched with the negative emotions in terms of their arousal.

## General Discussion

The primary aim of the present study was to investigate the relative role of arousal and valence of emotion in determining the rate of impairment in EIB. We hypothesized that if arousal is critical for the impairment then any stimuli, which are high in arousal, will elicit EIB irrespective of its affective valence. In the first experiment, we used two categories of emotional distractor images that had on average similar affective valence but were with different levels of arousal. Keeping one factor constant, while varying the other, allows us to objectively examine whether EIB is modulated by the arousal level or by valence of the emotional image. Experiment 1 showed that despite of the fixed valence, stimuli high in arousal level causes more impairment as compared to low arousal stimuli. In Experiment 2, we kept the arousal level constant while varying the valence. The results of Experiment 2 showed that both positive and negative distractor images causes greater impairment in target identification as compared to neutral distractor images.

The importance of understanding the differential role of both arousal and valence in processing is steadily gaining prominence and is relatively undisputed in the recent times. A meta-analytical study done by Lundqvist et al. (2013), examining the role of emotional arousal and valence in visual search emphasizes that both valence and arousal should be accounted for while examining the role of emotion on perception. In their study, they have shown that contradictory findings (anger superiority effect and happy superiority effect) observed in previous studies can be easily explained by the differences in the arousal level of the stimuli. They also argue that giving more emphasis to the valence compared to arousal could be problematic while trying to understand the interaction between cognition and emotion.

A better understanding of EIB can be gained by looking at the theoretical account that explains the impairment in EIB. According to Most and colleagues (Most and Wang, 2011; Kennedy and Most, 2012; Wang et al., 2012) EIB is caused by an impairment in early level perceptual processing caused by the emotionality of the distractor image. According, to their Spatio-temporal Competition Account, two stimuli that appear in close spatial and temporal proximity competes for processing and the salient emotional stimuli wins at the cost of the non-salient neutral target. It is not clear how spatio-temporal competition explains the differential sensitivity of arousal and valence in determining EIB. An alternate recent account that explains a range of effects associated with the processing of emotional stimuli is the ABC model (Mather and Sutherland, 2011).

Arousal Biased Competition theory suggests that arousal biases the competition by improving perception of the high priority stimuli and weakening the perception of low priority stimuli. According to them, a very critical component in determining the priority is arousal. Specifically, if the emotional stimulus is high in arousal level, they get competitive advantage over stimuli with lower arousing level. They suggest that this prioritized processing of arousing images is due to the increased amygdalar activation during the processing of these stimuli and the influence of the amygdala activation on the fronto-parietal attentional network. This modulation of amygdala on fronto-parietal attentional network can be seen even if the emotionally arousing image is irrelevant for the task. That is, there are two ways through which amygdala modulates the fronto-parietal activity: first by enhancing the activation of the fronto-parietal network for the goal relevant arousing stimuli (facilitation of the emotional stimulus); second, by inhibiting the fronto-parietal activation in the presence of emotionally irrelevant stimuli, leading to the prioritized processing of the arousing stimuli (impairment in processing of the subsequent neutral stimulus). Importantly, many researchers have found that amygdala activity is sensitive to the arousal of the stimuli irrespective of their valence (Anderson et al., 2003; Cunningham et al., 2004).

Recently, in an EEG ERP study, Kennedy et al. (2014) showed that EIB shows the same ERP markers as AB, suggesting that the mechanisms involved in AB and EIB are similar. They call for further investigations to show that these overlaps indicate that both AB and EIB are very similar mechanism. The current paper offers further support for this claim by showing that theoretically, the biased competition model that explains AB can also account for EIB without making further assumptions about special processing of the emotional valence of the stimulus. Moreover, it also clearly refutes the claim that valence plays a special and central role in the manifestation of EIB.

## Ethics Statement

This study was carried out in accordance with the recommendations of IIT Gandhinagar Institutional Ethics Committee with written informed consent from all subjects. All subjects gave written informed consent in accordance with the Declaration of Helsinki. The protocol was approved by the Technical Committee, IITGN IEC.

## Author Contributions

DS was involved in the study design, data collection, analysis and writing the first draft of the paper. MS was involved in the study design, analysis and finalized the draft for publication.

## Conflict of Interest Statement

The authors declare that the research was conducted in the absence of any commercial or financial relationships that could be construed as a potential conflict of interest. The reviewer AC and handling Editor declared their shared affiliation, and the handling Editor states that the process nevertheless met the standards of a fair and objective review.

## Footnotes

1 In recent years though, reserchers have shown similar neurophysiological markers for both AB and EIB. That is, Kennedy et al. (2014) showed that P3b component which is responsible for WM consolidation is observed in both AB and EIB.

2 The term arousal level refers to the subjective arousal level.

## References

[B1] Alpers G. W. Gerdes A. B. M. (2007). Here is looking at you: emotional faces predominate in binocular rivalry. *Emotion* 7 495–506. 10.1037/1528-3542.7.3.495 17683206

[B2] Anderson A. K. Christoff K. Stappen I. Panitz D. Ghahremani D. G. Glover G. (2003). Dissociated neural representations of intensity and valence in human olfaction. *Nat. Neurosci.* 6 196–202. 10.1038/nn1001 12536208

[B3] Arnell K. M. Killman K. V. Fijavz D. (2007). Blinded by emotion: target misses follow attention capture by arousing distractors in RSVP. *Emotion* 7 465–477. 10.1037/1528-3542.7.3.465 17683203

[B4] Brainard D. H. (1997). The psychophysics toolbox. *Spat. Vis.* 10 433–436. 10.1163/156856897X00357 9176952

[B5] Bublatzky F. Gerdes A. B. M. White A. J. Riemer M. Alpers G. W. (2014). Social and emotional relevance in face processing: happy faces of future interaction partners enhance the late positive potential. *Front. Hum. Neurosci.* 8:493 10.3389/fnhum.2014.00493 PMC4100576 25076881

[B6] Carretié L. Hinojosa J. A. Martín-Loeches M. Mercado F. Tapia M. (2004). Automatic attention to emotional stimuli: neural correlates. *Hum. Brain Mapp.* 22 290–299. 10.1002/hbm.20037 15202107 PMC6871850

[B7] Ciesielski B. G. Armstrong T. Zald D. H. Olatunji B. O. (2010). Emotion modulation of visual attention: categorical and temporal characteristics. *PLoS ONE* 5:e13860 10.1371/journal.pone.0013860 PMC2974644 21079773

[B8] Cunningham W. A. Raye C. L. Johnson M. K. (2004). Implicit and explicit evaluation: FMRI correlates of valence, emotional intensity, and control in the processing of attitudes. *J. Cogn. Neurosci.* 16 1717–1729. 10.1162/0898929042947919 15701224

[B9] Kennedy B. L. Most S. B. (2012). Perceptual, not memorial, disruption underlies emotion-induced blindness. *Emotion* 12 199–202. 10.1037/a0026380 22148991

[B10] Kennedy B. L. Most S. B. (2015a). Affective stimuli capture attention regardless of categorical distinctiveness: an emotion-induced blindness study. *Vis. Cogn.* 23 105–117. 10.1080/13506285.2015.1024300

[B11] Kennedy B. L. Most S. B. (2015b). The rapid perceptual impact of emotional distractors. *PLoS ONE* 10:e0129320 10.1371/journal.pone.0129320 PMC4468095 26075603

[B12] Kennedy B. L. Rawding J. Most S. B. Hoffman J. E. (2014). Emotion-induced blindness reflects competition at early and late processing stages: an ERP study. *Cogn. Affect. Behav. Neurosci.* 14 1485–1498. 10.3758/s13415-014-0303-x 24897955

[B13] Kensinger E. A. (2004). Remembering emotional experiences: the contribution of valence and arousal. *Rev. Neurosci.* 15 241–253. 10.1515/REVNEURO.2004.15.4.241 15526549

[B14] Kensinger E. A. (2009). Remembering the details: effects of emotion. *Emot. Rev.* 1 99–113. 10.1177/1754073908100432 19421427 PMC2676782

[B15] Kensinger E. A. Corkin S. (2004). Two routes to emotional memory: distinct neural processes for valence and arousal. *Proc. Natl. Acad. Sci. U.S.A.* 101 3310–3315. 10.1073/pnas.0306408101 14981255 PMC365786

[B16] Kensinger E. A. Schacter D. L. (2006). Processing emotional pictures and words: effects of valence and arousal. *Cogn. Affect. Behav. Neurosci.* 6 110–126. 10.3758/CABN.6.2.110 17007232

[B17] Lang P. J. Bradley M. M. (2010). Emotion and the motivational brain. *Biol. Psychol.* 84 437–450. 10.1016/j.biopsycho.2009.10.007 19879918 PMC3612949

[B18] Lang P. J. Bradley M. M. Cuthbert B. N. (2008). *International Affective Picture System (IAPS): Affective Ratings of Pictures and Instruction Manual.* Technical Report A-8. Gainesville, FL: University of Florida.

[B19] LeDoux J. (1996). *The Emotional Brain: The Mysterious Underpinnings of Emotional Life.* New York, NY: Simon and Schuster.

[B20] Lundqvist D. Juth P. Ohman A. (2013). Using facial emotional stimuli in visual search experiments: the arousal factor explains contradictory results. *Cogn. Emot.* 28 1012–1029. 10.1080/02699931.2013.867479 24341823

[B21] Mather M. Sutherland M. R. (2011). Arousal-biased competition in perception and memory. *Perspect. Psychol. Sci.* 6 114–133. 10.1177/1745691611400234 21660127 PMC3110019

[B22] Mathewson K. J. Arnell K. M. Mansfield C. A. (2008). Capturing and holding attention: the impact of emotional words in rapid serial visual presentation. *Mem. Cogn.* 36 182–200. 10.3758/MC.36.1.182 18323074

[B23] McConnell M. M. Shore D. I. (2011). Upbeat and happy: arousal as an important factor in studying attention. *Cogn. Emot.* 25 1184–1195. 10.1080/02699931.2010.524396 22017613

[B24] Most S. B. Chun M. M. Widders D. M. Zald D. H. (2005). Attentional rubbernecking: cognitive control and personality in emotion-induced blindness. *Psychon. Bull. Rev.* 12 654–661. 10.3758/BF03196754 16447378

[B25] Most S. B. Jungé J. A. (2008). Don’t look back: retroactive, dynamic costs and benefits of emotional capture. *Vis. Cogn.* 16 262–278. 10.1080/13506280701490062

[B26] Most S. B. Smith S. D. Cooter A. B. Levy B. N. Zald D. H. (2007). The naked truth: positive, arousing distractors impair rapid target perception. *Cogn. Emot.* 21 964–981. 10.1080/02699930600959340

[B27] Most S. B. Wang L. (2011). Dissociating spatial attention and awareness in emotion-induced blindness. *Psychol. Sci.* 22 300–305. 10.1177/0956797610397665 21270446

[B28] Öhman A. (1986). Face the beast and fear the face: animal and social fears as prototypes for evolutionary analyses of emotion. *Psychophysiology* 23 123–145. 10.1111/j.1469-8986.1986.tb00608.x 3704069

[B29] Öhman A. Flykt A. Esteves F. (2001). Emotion drives attention: detecting the snake in the grass. *J. Exp. Psychol. Gen.* 130 466–478. 10.1037/0096-3445.130.3.466 11561921

[B30] Panksepp J. (1998). *Affective Neuroscience: The Foundations of Human and Animal Emotions.* New York, NY: Oxford University Press.

[B31] Pelli D. G. (1997). Pixel independence: measuring spatial interactions on a CRT display. *Spat. Vis.* 10 443–446. 10.1163/156856897X00375 9176954

[B32] Phelps E. A. Ling S. Carrasco M. (2006). Emotion facilitates perception and potentiates the perceptual benefits of attention. *Psychol. Sci.* 17 292–299. 10.1111/j.1467-9280.2006.01701.x 16623685 PMC1555625

[B33] Pool E. Brosch T. Delplanque S. Sander D. (2015). Attentional bias for positive emotional stimuli: a meta-analytic investigation. *Psychol. Bull.* 142 79–106. 10.1037/bul0000026 26390266

[B34] Pratto F. John O. (1991). Automatic vigilance: the attention-grabbing power of negative social information. *J. Pers. Soc. Psychol.* 61 380–391. 10.1037/0022-3514.61.3.380 1941510

[B35] Raymond J. E. Shapiro K. L. Arnell K. M. (1992). Temporary suppression of visual processing in an RSVP task: an attentional blink? *J. Exp. Psychol.* 18 849–860. 10.1037/0096-1523.18.3.849 1500880

[B36] Schimmack U. Derryberry D. (2005). Attentional interference effects of emotional pictures: threat, negativity, or arousal? *Emotion* 5 55–66. 15755219 10.1037/1528-3542.5.1.55

[B37] Smith S. D. Most S. B. Newsome L. A. Zald D. H. (2006). An emotion-induced attentional blink elicited by aversively conditioned stimuli. *Emotion* 6 523–527. 10.1037/1528-3542.6.3.523 16938093

[B38] Taylor S. E. (1991). Asymmetrical effects of positive and negative events: the mobilization-minimization hypothesis. *Psychol. Bull.* 110 67–85. 10.1037/0033-2909.110.1.67 1891519

[B39] Verbruggen F. De Houwer J. (2007). Do emotional stimuli interfere with response inhibition? Evidence from the stop signal paradigm. *Cogn. Emot.* 21 391–403. 10.1080/02699930600625081

[B40] Vuilleumier P. Armony J. L. Driver J. Dolan R. J. (2001). Effects of attention and emotion on face processing in the human brain: an event-related fMRI study. *Neuron* 30 829–841. 10.1016/S0896-6273(01)00328-2 11430815

[B41] Wang L. Kennedy B. L. Most S. B. (2012). When emotion blinds: a spatiotemporal competition account of emotion-induced blindness. *Front. Psychol.* 3:438 10.3389/fpsyg.2012.00438 PMC3491583 23162497

[B42] Watson D. G. Blagrove E. Evans C. Moore L. (2012). Negative triangles: simple geometric shapes convey emotional valence. *Emotion* 12 18–22. 10.1037/a0024495 21787078

[B43] Watson D. G. Blagrove E. Selwood S. (2011). Emotional triangles: a test of emotion-based attentional capture by simple geometric shapes. *Cogn. Emot.* 25 1149–1164. 10.1080/02699931.2010.525861 21432646

[B44] Yang Q. Wang X. Yin S. Zhao X. Tan J. Chen A. (2016). Improved emotional conflict control triggered by the processing priority of negative emotion. *Sci. Rep.* 6:24302 10.1038/srep24302 PMC4834577 27086908

[B45] Yiend J. (2010). The effects of emotion on attention: a review of attentional processing of emotional information. *Cogn. Emot.* 24 3–47. 10.1080/02699930903205698

